# News and Coming Events

**Published:** 1908-04-04

**Authors:** 


### NEWS AND COMING EVENTS.

Dr. W. Wright succeeds Dr. Arthur Keith as senior demonstrator of anatomy at the London Hospital.

The new buildings of the College of Physicians of Philadelphia are to be erected shortly at an estimated cost of $300,000.

Mr. Howard Russell, M.S., F.R.C.S., has been appointed curator of the Museum of the London Hospital Medical College.

Dr. G. F. Darwall Smith, F.R.C.S., has been appointed a physician to out-patients at the British Lying-in Hospital, Endell Street, W.C.

At the University of Oxford H. R. Dean, B.M., B.Ch., New College, M.R.C.P., formerly Senior Demy of Magdalen College, has been elected to a Radcliffe Travelling Fellowship for three years.

Sir Felix Semon has been appointed by the Royal College of Physicians of London to represent the College at the International Congress on Laryngology and Rhinology, to be held at Vienna on April 21 to 28.

Sir Henry Littlejohn, M.D., LLD., has resigned the post of Medical Officer of Health for Edinburgh, after nearly fifty years' service in that capacity.

The thirtieth ordinary general meeting of the Home Hospitals Association for Paying Patients was held at Fitzroy House, London, W., on Monday, March 30, under the chairmanship of H. G. Hammersley, Esq.

At a meeting of the Senate of the University of London, held on March 25, Dr. St. Clair Thompson was appointed to represent the University at the International Laryngo-Rhinological Congress, to be held at Vienna this month.

M. Paul Cambon, Ambassador of France, will preside at the fortieth annual banquet of the French Hospital and Dispensary, Shaftesbury Avenue, on May 16, at the Hôtel Cecil. The Lord Mayor and the Sheriffs will be present.

Mr. Rickman J. Godlee, F.R.C.S., has been elected as the representative of the Royal College of Surgeons of England on the Council of University College, Bristol, to fill the vacancy caused by the resignation of Mr. Thomas Bryant.

Dr. Arthur Newsholme, who has recently been appointed principal medical officer to the Local Government Board, was entertained at a banquet at the Royal Pavilion, Brighton, on March 21 as a complimentary recognition by the inhabitants of his valuable services as medical officer of health for the borough for nearly twenty years.

The Governors of the Isle of Man Hospital have accepted an offer from Noble's Trustees to give £20,000 for the erection of a new hospital, and also a site, and to endow the hospital, when completed, with another £20,000. The offer was conditional on the present buildings being handed over to the trustees free from any debt.

In order to celebrate the distinction lately conferred upon him by the King, Sir Malcolm Morris, K.C.V.O., was entertained at dinner at the Imperial Restaurant, on March 19, by a number of friends, many of them also distinguished in the field of dermatology. Dr. Radcliffe Crocker was in the chair. Reference was made by him and others to the long and valuable services rendered by Sir Malcolm Morris to the science of dermatology and to the literature of medicine generally.

To fill the place on the General Medical Council rendered vacant by the death of Sir Thomas McCall Anderson, the University Court of the University of Glasgow has appointed Principal MacAlister as their representative for the next five years.

The Royal Commission on Vivisection held meetings on March 18 and 25, when the witnesses were Lieut.-Col. Lawrie, Dr. L. E. Shore, Mr. W. Hall, and Dr. H. Anderson, of the Physiological Laboratory, Cambridge. The Commission then finally decided to close their evidence and proceed to the consideration of their report.

Surgeon-General Walter Wyman, of the U.S.A. Public Health Service, has submitted a Report on "Milk in its Relation to Public Health," in which he says that out of 500 fever epidemics, definitely traced to the milk supplies of the affected districts, 317 were enteric fever, 125 scarlet fever, 51 diphtheria, and 7 were pseudo-diphtheria epidemics.

A Civil List pension of £120 per annum has been granted by his Majesty, on the advice of the Prime Minister, to Dr. J. Hall Edwards, of Birmingham, whose recent disablement in the cause of medical science has attracted much sympathetic attention. The treasurer of the local supplementary fund is Mr. F. Marsh, 95 Cornwall Street, Birmingham, to whom subscriptions should be sent.

The King has been pleased to give to Arthur Ferguson MacCallan, M.B., F.R.C.S., chief ophthalmic inspector in the Public Health Department of the Egyptian Government, his Majesty's license and authority to accept the Insignia of the Third Class of the Imperial Ottoman Order of the Medjidieh, conferred upon him by the Khedive of Egypt, authorised by the Sultan of Turkey, in recognition of valuable services rendered by him.

The annual meeting of the Royal Dental Hospital, Leicester Square, was held on March 26, the Hon. W. F. D. Smith, M.P., in the chair. The Committee of Management reported that legacies of £1,000 for general purposes and £1,000 to found a scholarship in dental surgery, to be called the "Alfred James Woodhouse Scholarship," had been bequeathed to the hospital by the late A. J. Woodhouse, Esq., a generous supporter, and formerly a vice-president of the hospital. During the present year the hospital completes its jubilee, and it has been decided to hold a festival dinner at the Hôtel Cecil on Thursday, June 25, at which Sir Frederick Treves, consulting surgeon to the hospital, will preside.

The annual general meeting of University College Hospital was held on March 26, under the presidency of Henry Lucas, Esq., Treasurer. The report for 1907 states that the Committee, owing to lack of means, have been unable to open a ward containing 24 beds, which should be available for the sick poor. This means that about 350 patients annually, many requiring immediate treatment in the wards, must be refused admission. An additional annual expenditure of about £1,200 will be required to keep the ward open, and the Committee have not felt justified in incurring this further liability. The upkeep of the hospital involves an annual cost of about £28,000, and the annual charge of nearly 3,300 poor sick people in the wards, and of over 50,000 out-patients suffering from accident or illness is a serious responsibility. In order to meet the deficiency in income the Committee have had to trench upon their small invested funds by the realisation of £1,500 Consols, and to devote to purposes of current expenditure, legacies which, in more prosperous times, could have been invested.

At a special meeting of the Governors of the North-Eastern Hospital for Children, held on April 2, the resolution of the General Committee to alter the name of the institution to "The Queen's Hospital for Children" was confirmed. As reported in this column last week Her Majesty's permission for this change of title has been recently obtained. It is further announced that for the purpose of freeing the Hospital from debt a Bazaar will be held in the Shoreditch Town Hall on June 23, 24 and 25, and will be opened by H.R.H. Princess Henry of Battenberg.

Arrangements are being made by the University of Cambridge to celebrate on June 22, 23 and 24, 1909, the 100th anniversary of the birth of Charles Darwin, and the 50th anniversary of the publication of the "Origin of Species." It is proposed to invite representatives of Universities and other learned bodies, together with distinguished individuals, to visit the University on the occasion. A programme of the celebration will shortly be issued. The Hon. Secretaries are Mr. J. W. Clark, Registrary, and Professor A. C. Seward, Professor of Botany in the University.

A special meeting of the Executive Committee of the Hospital Saturday Fund was held last month, to receive the resignation of Mr. H. N. Hamilton-Hoare, who has been the honorary treasurer of the fund since its establishment in 1873. The resignation was received with profound regret, and the Committee expressed their sincere thanks to Mr. Hoare for all that he had done for the fund, and their hope that he would retain the position of Vice-President. The Committee has approached the Hon. H. L. W. Lawson, who has consented to accept the hon. treasurership of the fund.

The authorities at St. Petersburg are taking precautions against any possible outbreak of cholera in St. Petersburg during the spring and summer. Should the disease appear arrangements have been made for dividing the city into 143 districts, each under the supervision of a medical officer. Numerous disinfecting stations will be established, and every householder will also be obliged to keep his own supply of disinfectants. The sanitary depots will all possess ambulances, and will be connected with each other by telephone. Popular instruction in methods of prevention and treatment will be provided.

